# Transformer model to determine spatio-temporal relationships of variables, and interpretability for soybean seed yield, oil, and protein prediction

**DOI:** 10.3389/frai.2026.1750108

**Published:** 2026-02-26

**Authors:** Timilehin T. Ayanlade, Liza Van der Laan, Qisai Liu, Tryambak Gangopadhyay, Johnathon Shook, Arti Singh, Baskar Ganapathysubramanian, Soumik Sarkar, Asheesh K. Singh

**Affiliations:** 1 Department of Mechanical Engineering, Iowa State University, Ames, IA, United States; 2 Department of Agronomy, Iowa State University, Ames, IA, United States; 3 Amazon Web Services, Amazon, Santa Clara, CA, United States; 4 Department of Computer Science, Iowa State University, Ames, IA, United States

**Keywords:** interpretability, seed oil, seed protein, soybean, spatio-temporal analysis, transformer, yield prediction

## Abstract

Accurate in-season prediction of seed yield and seed composition traits such as oil and protein are useful for gaining accuracy and efficiency in soybean breeding. These predictions can also inform farmers, enabling them to improve their field management practices, and guide their market decisions. We report a Transformer-based deep learning framework built on 30 years of multi-environment performance data from the Northern and Southern Uniform Soybean Tests (UST) across North America. Unlike earlier studies on seed yield, oil and protein prediction that focus on limited years, regions, single modalities, we utilized a comprehensive dataset that includes weather, genotype, and management factors, ensuring a more holistic approach to soybean yield, oil, and protein prediction. Our model integrates multivariate time-series weather data with genotypic relationship information, maturity group, and geographic location, to predict variety performance in diverse environments. Our model captures complex temporal patterns associated with trait variability; showing high predictive accuracy (R2) of 77.6 ± 0.2%, 63.9 ± 4.7%, and 79.3 ± 2.3% for seed yield, oil, and protein, respectively. Additionally, for seed yield, we also evaluated multiple interpretability methods to assess feature importance for predictor variables and critical growing timepoints, and solar radiation and temperature were noted as the key predictors. Overall, these results demonstrate the usefulness of a Transformer-based model in trait predictions, and the utility of large cooperative datasets from breeding programs.

## 1 Introduction

Predicting cultivar performance with high accuracy and interpretability is essential for optimizing plant breeding strategies and mitigating the risks posed by climate variability. Crop performance, such as yield, is influenced by complex interactions between genetic factors and environmental conditions (GxE), making it challenging to develop reliable predictive models. The prediction of crop performance in untested environments is an enduring challenge in plant breeding, and is an active area of research with numerous approaches being investigated, such as genomic prediction (Meuwissen et al., 2001; Jannink et al., 2010; Tolhurst et al., 2022), phenomic prediction (Parmley et al., 2019; Winn et al., 2023; Singh et al., 2021; Van der Laan et al., 2025), and crop modeling (Jagtap and Jones, 2002; Messina et al., 2006; Sakamoto, 2020), as well as the integration of these different methods (Technow et al., 2015; Cooper et al., 2020; González et al., 2024; Crossa et al., 2025). Traditional crop modeling approaches, including process-based and statistical models, have been widely used but often require extensive datasets and struggle to capture the intricate, non-linear relationships between variables (Jones et al., 2003; Boote et al., 1996).

Machine learning (ML), particularly deep learning, offers an alternative for yield prediction but has historically faced limitations in interpretability, which is critical for domain experts making breeding and management decisions (Ashfaq et al., 2025; Mahesh and Soundrapandiyan, 2024). Additionally, training ML models requires extensive datasets, which are often costly and time-consuming to generate.

Soybean is one of the most grown crops in North America, and breeding efforts have been extensive for the crop. As part of the breeding efforts for North American soybeans, the annual Uniform Soybean Tests (UST) have been coordinated through the United States Department of Agriculture (USDA) since 1941 (USDA-ARS, 2025a,b). Starting out as yield trials initially, the UST is a partnership with public breeders in universities and government organizations across the US and Canada. These trials are meant to allow public breeding programs and small private programs to test current and experimental varieties in a greater number of new environments within the intended range of adaptation. This allows public breeding programs to expand the area of testing available with less associated costs, and also serve as a valuable source of historical and current soybean data. Because these data are publicly available, they are an affordable option for research in predicting crop performance (Krause et al., 2023). Such large collaborative networks involving multiple breeders and researchers generating large multi-year, multi-environment datasets has been utilized for comprehensive studies (Shook et al., 2021; Wartha et al., 2025; Chiozza et al., 2025), and these valuable datasets provide tremendous opportunities for ML-based model development for agriculture and breeding applications.

Recent advances in attention-based models, particularly transformers, have demonstrated significant improvements in time-series prediction by efficiently capturing long-range dependencies and highlighting critical input features (Vaswani et al., 2017; Singh et al., 2025). Unlike long short-term memory models (LSTMs) (Hochreiter and Schmidhuber, 1997), which process sequences sequentially, transformers utilize self-attention mechanisms to weigh the importance of different time steps and variables simultaneously. This makes them particularly well-suited for yield prediction, where weather variability across the growing season plays a crucial role in determining crop performance (Tan et al., 2018; Shook et al., 2021). Compared to traditional statistical models and deep learning methods such as LSTMs, transformers offer a more flexible and scalable framework for handling complex, multivariate agricultural datasets. The ability to process entire sequences in parallel allows for efficient learning of patterns across years and locations (Shi et al., 2024). Furthermore, these models improves interpretability by explicitly highlighting key weather variables and their temporal importance, aiding plant breeders and agronomists in understanding the drivers of yield variability. Prior studies have utilized traditional and deep learning techniques to model temporal dependencies in yield prediction, there is a need to understand the role(s) of environmental factors and time periods that most influence the critical traits (Lobell et al., 2013), such as soybean seed yield, oil, and protein.

Interpretability plays a vital role in yield prediction by aligning model outputs with agronomic expertise and supporting decision making in breeding programs. This need becomes even more pressing in multi-sensor or multi-source settings, where interpretability has often been cited as a significant gap in current approaches (Ayanlade et al., 2024; Singh et al., 2025). Prior studies have explored both intrinsic and *post-hoc* interpretability approaches. Intrinsic methods, such as linear regression and decision trees (Monteiro et al., 2022; Dwivedi et al., 2023; Balcan and Sharma, 2024; Torsoni et al., 2023), directly expose feature contributions through their model structures, while *post-hoc* methods—such as LIME (Ribeiro et al., 2016), SHAP (Lundberg and Lee, 2017), Integrated Gradients (Sundararajan et al., 2017), and DeepLIFT (Shrikumar et al., 2017) approximate local explanations for complex deep models. In recent years, attention-based architectures have also gained prominence as interpretable modeling strategies for sequential and multimodal data, particularly in crop monitoring and yield prediction tasks where temporal attention highlights critical growth stages or stress periods (Xiong et al., 2024; Aviles Toledo et al., 2024; Shook et al., 2021; Wang et al., 2024). However, both intrinsic and *post-hoc* methods often lack standardized measures of faithfulness, raising concerns about whether their explanations truly reflect the model's decision process rather than coincidental correlations. To address this limitation, evaluation pipelines based on quantitative faithfulness metrics, such as comprehensiveness and sufficiency (DeYoung et al., 2019), and their regression-oriented extension AOPCR (Ozyegen et al., 2022) have been established to assess how model predictions respond to systematic perturbations of the most relevant features. These frameworks enable rigorous evaluation of interpretability methods across domains, providing deeper insight into the reliability of attention- and attribution-based explanations in multi-source agronomic modeling.

In this study, we propose a Transformer-based deep learning model for the prediction of soybean seed yield, oil and protein using historical data from the Uniform Soybean Trials (UST), encompassing the Northern and Southern trials. Our model integrates multidecadal weather records with pedigree information, leveraging recent advances in sequence modeling to improve both predictive accuracy and model interpretability. In addition, by leveraging the self-attention mechanism of Transformer architectures, we identify the most influential time periods and variables during the growing season, providing valuable insights into how environmental factors impact yield, oil and protein levels, offering a valuable tool to potentially mitigate risks and improve agricultural and breeding decision-making. These insights will inform breeding strategies aimed at developing resilient cultivars, ultimately contributing to global food security (Rose et al., 2016). Previous studies that applied attention-based mechanisms to yield prediction (Xiong et al., 2024; Aviles Toledo et al., 2024; Shook et al., 2021), primarily relied on LSTMs and meteorological data without integrating genetic information and crop management practices including pre-planting information. Our work bridges this gap by incorporating a comprehensive dataset including weather, genotype, and management factors, with Transformer-based modeling ensuring a more holistic approach to soybean yield, oil, and protein prediction.

## 2 Materials and methods

### 2.1 Data preparation and processing

#### 2.1.1 Performance records

PDF files from the 1989-2018 Uniform Soybean Tests (USTs) (USDA-ARS, 2025a,b) were downloaded. Following the protocol outlined in Shook et al. (2021), all PDFs were converted to .xlsx files. Briefly, the on-line utility Zamzar (zamzar.com) for conversions to .xlsx files. Each tab of the resulting .xlsx files correspond to a single page from the original PDF file. This methodology allows for the recovery of the PDF tables with no need for error-prone human translation. Random checking of files for errors was conducted to ensure the accuracy of the data, and no instance of error was noticed. Once in .xlsx files, the tables were manually sorted to align the performance records for a given genotype-environment combination into a single row. Records with no yield data were removed from the file to ensure data integrity. To standardize the dataset, performance records were curated such that each genotype-location-year combination was consolidated into a single row. Following data cleaning, the final dataset comprised 103,955 performance records over 29 years, with 11,506 unique genotypes. Each performance record contains 214 days of multivariate time-series data, corresponding to the growing season within the year, with each record characterized by the maturity group variables.

#### 2.1.2 Acquisition of weather records

Daily weather records for all year/location combinations were compiled from NASA Power (NASA, 2025) and using the closest grid point from the 1/2° × 5/8° global grid utilized by the MERRA-2 model implemented within NASA Power. The weather data included the maximum, minimum, and average air temperature (°C), relative humidity (percentage), average precipitation (mm day^−1^), and total solar irradiance (MJ/m^2^). For model training, we downsampled the dataset to extract maximum, minimum, and average weather conditions over different time intervals throughout the growing season. This processed weather information was then concatenated with the performance records to create a complete dataset for prediction. Notably, the available performance records for oil and protein had missing records for the combined data (49.3% of total records missing), and motivated our exploration of transfer learning strategies. The missing records for oil and protein were environment specific, and are due to only a subset of environments in the UST network providing these trait data.

#### 2.1.3 Genotype clustering

Genotype identity was preserved in the performance records so that it could be applied to the model and allow for prediction of specific genotypes. The UST program was used so that experimental genotypes can be tested on a large scale. Because of this, most of the genotypes are experimental lines which do not have molecular marker data available. Additionally, this dataset extends into a time when genotyping was cost prohibitive for many programs, further limiting the availability of molecular marker data from the older genotypes. While there are recent efforts to genotypes all lines, gaps still persists. Additionally, we wanted to explore the usefulness for crops or in situation where marker data are not available. This prevents the use of a G matrix for use in genotype clustering. As an alternative to these restrictions, we developed a connected pedigree for all genotypes that had publicly available pedigree information. This allowed for the creation of an A matrix, using the pedigree package in R. The A matrix then resulted in the formation of a 11,506 × 11,506 kinship matrix. The kinship matrix and genotype program of origin were used together to increase control over relatedness. This allowed for genotype clustering, which was found to improve model performance in Shook et al. (2021).

To incorporate genotype-specific variation into the model, we applied clustering techniques based on the A matrix of kinship relationships and mean location yield across genotypes. Using the K-means clustering approach, we determined that the optimal number of clusters was 40. Details are provided in Supplementary material 1. This allowed genotypes to be assigned to clusters based on the correlation matrix, effectively grouping lines with similar genetic backgrounds. K-means clustering partitions data into a predefined number of clusters (n) by iteratively minimizing intra-cluster variance. Specifically, the algorithm identifies n cluster centroids and assigns each genotype to the nearest centroid, minimizing the within-cluster sum-of-squares (inertia). This method has demonstrated effectiveness in handling large datasets and is commonly applied across various scientific domains (Shook et al., 2021). Each genotype was assigned a fixed cluster ID, which was subsequently incorporated into our model as an additional feature to enable the model capture genetic variation for yield prediction. All clustering analyses were implemented in Python 3.8 using the scikit-learn (v1.2) library, with NumPy, Pandas, and SciPy for data preprocessing and matrix computation.

### 2.2 Model

**Figure 1:**
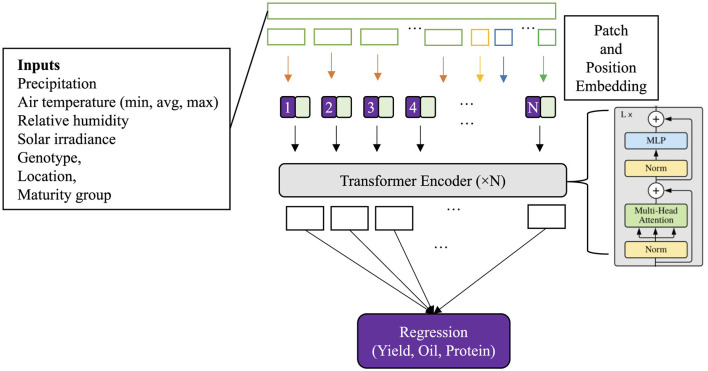
Architecture of the transformer model used for multi-trait prediction in soybean. Input features, including weather variables, genotype, maturity group, and location information, are segmented and embedded with patch and positional encodings. These are passed through a transformer encoder (4 encoder blocks) consisting of stacked layers of multi-head self-attention and feedforward neural networks. The model outputs trait predictions (yield, oil, and protein) through a regression head.

Our approach employs a transformer encoder model for yield prediction, leveraging self-attention mechanisms to capture dependencies across time steps in parallel. The prediction task is framed as a many-to-one regression problem where the input consists of time-series weather data alongside genotype and agronomic variables. Each training sample comprises a multivariate time series, containing weather-related variables and other context variables. To structure the input for the transformer model, we constructed tokens by aggregating daily weather variables into multiple day segments (see Figure 1), forming input tokens. The initial tokens for each variable were padded with zeros where necessary to maintain uniform sequence lengths. Additionally, the context tokens (genotype cluster, maturity group, and location—latitude and longitude) were also tokenized individually and added to the sequence.

As shown in Figure 1, each input input sequence


$$\begin{array}{l} x=[x_{hum1},x_{hum2},\ldots x_{temp1},x_{temp2},\ldots ,x_{rad1},x_{rad2},\ldots ,\\ \;x_{var*tp},x_{loc},x_{geno},x_{mg}],\;x\in {\mathbb{R}}^{T\times d} \end{array}$$

where *T* is the number of tokens, *d* is the dimensionality, *hum* represents humidity, *temp* represents the temperature related variables, *rad* represents radiation, *var* represents each weather variable, *tp* represents the token timepoint, *loc* represents latitude and longitude token, *geno* represents genotype, and *mg* represents the maturity group. Each token is embedded and positionally encoded:


$${\text{z}}_{i}^{\left(0\right)}=\text{E}{\text{x}}_{i}+{\text{p}}_{i}$$

where **E** ∈ ℝ^*d*×*d*^′^^ is a learnable projection matrix, and ${\text{p}}_{i}\in {\mathbb{R}}^{d^{\prime }}$ is the positional encoding for token *i*.

Similar to (Woo et al., 2024), the embedded sequence for all variables ${\text{z}}^{\left(0\right)}=\left[{\text{z}}_{1}^{\left(0\right)},\ldots ,{\text{z}}_{T}^{\left(0\right)}\right]$ is concatenated and passed through a unified encoder consisting of *N* stacked transformer encoder layers. Each layer consists of a multi-head self-attention module followed by a feedforward network.

The final token representations **z**^(*N*)^ ∈ ℝ^*T*×*d*^′^^ are flattened and passed to a regression head:


$$\hat{\text{y}}={\text{W}}_{\text{reg}}\cdot \text{concat}\left({\text{z}}^{\left(N\right)}\right)+{\text{b}}_{\text{reg}},\;\hat{\text{y}}\in \mathbb{R}$$

The model is trained using mean squared error (MSE) loss


$$L=\frac{1}{n}∥ŷ-y{∥}^{2}$$

where *y* is the ground truth value for desired trait and *n* is the number of samples.

For yield prediction, we trained the model end-to-end using a regression head applied to the flattened encoder outputs. In contrast, oil and protein prediction tasks had fewer available training samples. To address this data limitation, we adopted a transfer learning approach by reusing the embedding and transformer encoder pre-trained on the yield task. Specifically, we evaluated two strategies: (i) end-to-end fine-tuning of the entire model on the limited oil and protein data, and (ii) freezing the pre-trained encoder weights and training only a new regression head, each for oil and protein. This setup enabled us to assess the effectiveness of transferring temporal and environmental representations learned from the yield task to lesser data traits, seed oil, and protein.

All analyses were implemented using PyTorch (v1.12) and Python 3.8, with dependencies including NumPy, Pandas, SciPy, scikit-learn, and PyYAML for data preprocessing and configuration handling. Experiments were conducted on the Iowa State University Nova High-Performance Computing (HPC) cluster, running Linux (CentOS 9 / Kernel 5.14.0) with job scheduling via Slurm. Each job was executed on a single NVIDIA A100-SXM4 GPU (80 GB memory, 6912 CUDA cores, Ampere architecture) and utilized 16 CPU threads out of a total of 64 physical (128 logical) cores available per node. All training was performed using mixed-precision computation (FP16) enabled through PyTorch AMP. Model training was managed and tracked using the Weights and Biases experiment manager, with random seeds fixed to five values (0–4) for reproducibility. Average wall-clock training time per model was approximately 200 min for pre-training and the same for finetuning.

### 2.3 Baseline models

To assess the effectiveness of the transformer model, we compared its performance against several baseline models: Support Vector Regression with Radial Basis Function Kernel (SVR-RBF), Least Absolute Shrinkage and Selection Operator (LASSO) regression, Bidirectional Long Short-Term Memory (BLSTM), and Bidirectional Recurrent Neural Networks (BRNN). Each baseline was trained using hyperparameter optimization to ensure fair comparison.

### 2.4 Model training and evaluation

The models were optimized using the Adam optimizer. The dataset was randomly split into 80% training, 10% validation, and 10% test sets. To prevent overfitting, a dropout of 0.1 was applied within the transformer layers. The model was trained for a maximum of 100 epochs with a batch size of 16. We include early stopping with a patience of 20 epoch to prevent overfitting and to ensure that the model is well trained. Each model was trained with a learning rate of 5 × 10^−6^, and cosine learning rate adjustment strategy.

Model performance was evaluated using three standard regression metrics: root mean squared error (RMSE), mean absolute error (MAE), and coefficient of determination (R-squared, *R*^2^) score.

### 2.5 Model interpretability

To analyze model interpretability, we employed *post-hoc* attention-based and attribution-based techniques on the trained transformer model. First, the self-attention maps were extracted from each encoder layer and head for all test set samples. Attention weights were normalized and mean aggregated across heads and layers in the encoder block to compute importance score. By aggregating attention scores across samples and across layers, we identified weather patterns that strongly influenced trait predictions. The attention scores were extracted from the trained model and visualized to assess their alignment with established domain principles in crop production. We compare the performance of the attention-based score with gradient-based attribution methods such as Integrated Gradients (Sundararajan et al., 2017), DeepLIFT (Shrikumar et al., 2017), Gradient SHAP (Lundberg and Lee, 2017), and LIME (Ribeiro et al., 2016). These methods quantify the marginal contribution of each input token to the model's predictions by computing local gradients or perturbation responses.

### 2.6 Methodology for faithfulness evaluation of interpretations

Evaluating interpretability in the absence of ground-truth explanations requires a quantitative framework that measures the internal consistency between the model's behavior and the importance assigned to its input features. Following the approach of Ozyegen et al. (2022), DeYoung et al. (2019), Islam et al. (2024), we assess local interpretability using perturbation-based metrics that quantify the degree to which identified important tokens truly influence model predictions. For each interpretability method, the token relevance scores produced by the explanation algorithm were normalized and ranked in descending order to determine the most informative input positions. A fixed set of perturbation levels *k* ∈ {5, 7.5, 10, 15, 25} was used to define the top percentage of relevant tokens.

For each perturbation level, two complementary evaluations were performed. In the comprehensiveness setting, the top-*k* most relevant tokens were masked from the input while keeping all other tokens unchanged, and the model's new output was compared to the original prediction. The intuition is that if the explanation method correctly identifies truly important tokens, removing them should substantially degrade the model's confidence or alter its prediction. In contrast, the sufficiency setting retains only the top-*k* tokens and masks the rest, measuring whether the selected subset alone contains enough information to reconstruct the model's original output. A smaller difference in this case implies higher sufficiency.

The difference between the model's unperturbed and perturbed predictions was measured using both mean absolute error (MAE) and mean squared error (MSE), averaged over all test samples. The comprehensiveness and sufficiency losses were then aggregated across all perturbation levels to compute the Area Over the Perturbation Curve for Regression (AOPCR). This metric integrates the change in model performance as progressively larger fractions of the most relevant tokens are removed or retained. A higher AOPCR for comprehensiveness indicates that masking top-ranked tokens leads to stronger degradation in predictions, suggesting higher faithfulness, whereas a lower AOPCR for sufficiency implies that the explanation method successfully identifies a minimal subset of features sufficient to reproduce the model's decision.

## 3 Results

### 3.1 Evaluation of model performance

#### 3.1.1 Yield prediction

We evaluated the performance of five models using weather variables as inputs, with results summarized in Table 1. Among traditional machine learning approaches, SVR-RBF outperformed LASSO. However, both were outperformed by deep learning models. Within the recurrent neural network family, the BLSTM achieved better results compared to the BRNN, though the differences in performance were marginal. The best performance was observed with Transformer-based models. Our proposed transformer model achieved the highest R^2^ score (0.776 ± 0.002), with the lowest RMSE and MAE across all tested models. This show the effectiveness of attention-based architectures to model complex temporal patterns in weather driven yield prediction, largely capturing the performance differences in performance between environments and years.

**Table 1:** Performance of different models for yield prediction.

**Model**	**R^2^ score**	**RMSE**	**MAE**
LASSO	0.732 ± 0.000	7.692 ± 0.000	5.832 ± 0.000
SVR-RBF	0.757 ± 0.000	7.319 ± 0.000	5.478 ± 0.000
BRNN	0.752 ± 0.011	7.393 ± 0.020	5.582 ± 0.154
BLSTM	0.754 ± 0.034	7.370 ± 0.147	5.562 ± 0.023
Transformer	**0.776** **±0.002**	**7.029** **±0.026**	**5.317** **±0.019**

Bold values indicate the best performance for each metric.

#### 3.1.2 Oil and protein prediction

**Figure 2:**
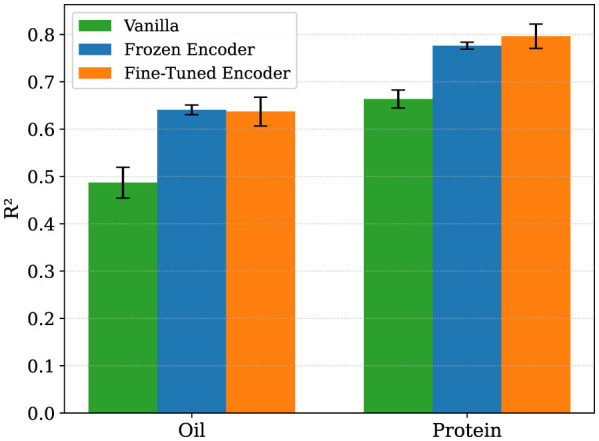
Comparison of performance of encoder training strategies (vanilla vs. frozen vs. fine-tuned) for oil and protein prediction. Bars show mean performance across five folds, with error bars representing standard deviation. Transfer learning strategies from the encoder pre-trained on yield consistently outperform direct finetuning for both traits, suggesting generalizability of the learned representations from yield. RMSE and MAE followed similar trends and are provided in the Supplementary material 11.

To assess the generalizability of representations learned during yield prediction, we evaluated two transfer learning strategies for oil and protein prediction, where we compared them to a baseline direct finetuning (vanilla) without pre-training (see Materials and Methods Section 2.2). First, we explore an end-to-end fine-tuning of a yield pre-trained Transformer model on oil and protein, and second, a variation where the encoder of the model pre-trained on yield is frozen, while training only the regression head on the target oil and protein trait. The results across five runs are shown in Figure 2.

For oil prediction, an end to end finetuning of the model resulted in the best performance, achieving a R^2^ score (63.9 ± 4.7%), with the freezing based transfer strategy achieving comparable performance. A similar trend was seen for protein, where full finetuning the encoder led to a notable gain in R^2^ (79.3 ± 2.3%), in comparison to a non pre-trained finetuning, and both models saw reduction in RMSE and MAE (see Supplementary material 5). These findings suggest that the pre-trained model had already captured generalizable temporal patterns relevant to other seed traits. End-to-end fine-tuning, while more flexible, may have suffered given the relatively limited dataset size. The results reinforce the value of leveraging pre-trained temporal encoders for downstream trait prediction, particularly when labeled data are limited.

### 3.2 Impact of temporal resolution on yield modeling

**Figure 3:**
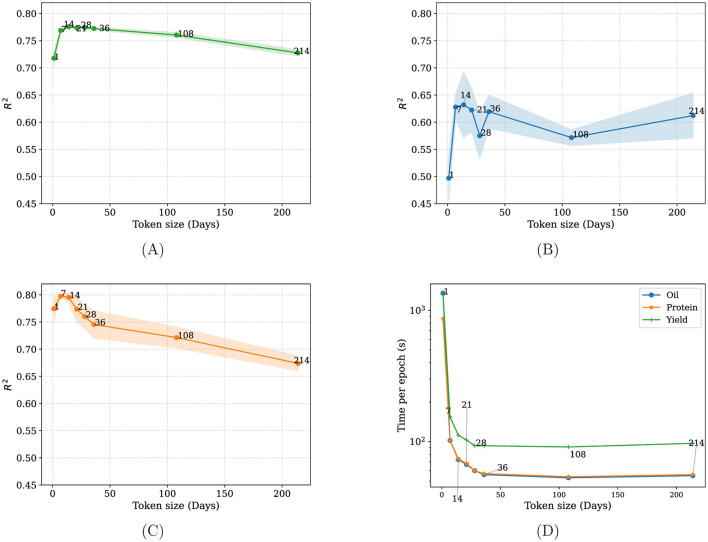
Effect of varying weather data sampling intervals on **(A)** yield **(B)** oil **(C)** protein **(D)** training time. Shaded regions show standard deviation across five runs.

To determine the optimal temporal resolution for encoding weather inputs, we evaluated model performance using different sampling intervals, by varying the number of days each input token represents. Prediction performance varied with the size of the temporal window, with the highest R^2^ observed at intermediate resolutions, a peak of 0.776 at a 14-day/tp sampling interval across all tasks (Figure 3).

Very fine grained sampling (e.g., daily tokens) led to slightly lower performance, likely due to increased sequence length, redundancy and extrapolation to the embedding dimension, which may complicate learning long-term dependencies. In contrast, overly coarse resolutions reduced temporal granularity, potentially omitting meaningful short-term weather fluctuations relevant to yield outcomes. The best performance across our test set was achieved using a 14 days per token, suggesting a balance between input compression and temporal fidelity.

### 3.3 Interpretability of yield model

In addition to yield prediction, our model provided insights about how much focus the model put on predictive variables for yield prediction across our test dataset. Following the methods described in Section 2.6, we apply various interpretability methods on our data using the trained model version on the 14-day/token sampling interval as it led to the best performance (see Figure 3), to investigate which methods show the best faithfulness in terms of comprehensiveness and sufficiency.

The attention-based method had the best faithfulness across both metrics, with the most comprehensiveness score and least sufficiency score (Table 2). Metrics for random samples in the Test set are provided in Supplementary material 8. We used the attention-based method for subsequent analysis in the following sections, as the overall results and randomly test samples consistently demonstrated that attention was among the top two methods.

**Table 2:** AOPCR-based faithfulness evaluation of interpretation methods averaged across test set.

**Method**	Comprehensiveness (↑)		Sufficiency (↓)	
	**MAE**	**MSE**	**MAE**	**MSE**
Gradient SHAP	0.29	3.49	0.36	5.13
Integrated Gradients	0.31	4.10	0.33	4.48
LIME	0.05	0.17	0.44	7.76
DeepLIFT	0.35	4.80	0.36	5.28
Attention	**0.39**	**6.12**	**0.28**	**3.53**

Higher values (↑) indicate better comprehensiveness; lower values (↓) indicate better sufficiency. Best results for each metric are shown in bold.

**Figure 4:**
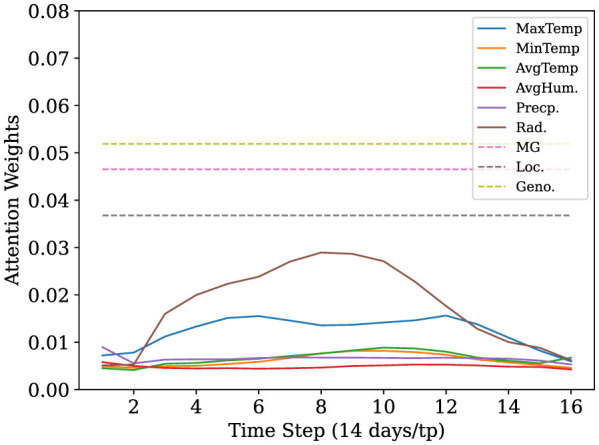
Average attention weights of yield predictive variables for the test set across five experimental runs for the 14 days per timepoint sampling intervals. Line plots represent the attention of time-varying variables over the growing season, while dashed horizontal lines represent static context variables such as maturity group (MG), location (Loc), and genotypic cluster (Geno).

Next, we analyzed the attention weights from the trained transformer model. This attention-based interpretability framework highlights tokens the model deems most relevant, based on the relationship to other tokens providing insight into genotype-environment interactions and the temporal dynamics underlying yield variability. Figure 4 illustrates the average attention weights assigned to individual weather variables throughout the growing season in our 14-day/tp sampling configuration, averaged across all samples in the test set. Notably, across all sampling intervals, we see that maximum temperature and solar radiation tokens received consistently high attention weights, aligning with established agronomic understanding of their influence on yield formation. The trend for the other sampling intervals across the test set and for two examples for random time-series samples in our test dataset are provided in Supplementary materials 9, 10, respectively. In our higher sampling fidelity models with 1-day/tp, we observe that maximum temperature tokens had the highest attention score (see Supplementary material 9).

**Figure 5:**
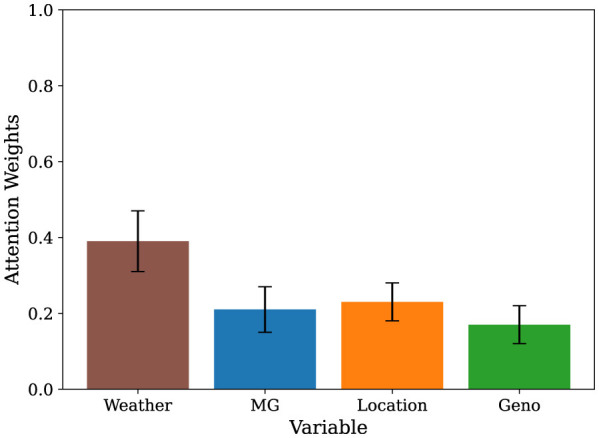
Average attention weights of yield predictive variables using transformer model. The weather bar represent the attention weight on the token when all weather variables (time-varying variables over the growing season) are treated as a single unit. The error bars show the standard error around the mean weights.

We also explored the quantification of the relative importance of dynamic weather features versus static context tokens, where we aggregated all time varying weather inputs into a single token and compared its average attention weight against the static variables, MG, genotype cluster, and location (see Figure 5). The composite weather token received the highest attention overall, with larger weights than any individual context. To verify that spatio-temporal features contribute meaningfully to prediction performance, we trained multiple model variants using (i) genetic features only, (ii) genetics combined with weather, and (iii) genetics combined with weather and management features. We observe a consistent improvement in performance as additional information is incorporated to the genetic features (see Supplementary material 12). This reinforces the central role of in season weather dynamics in yield determination, though also shows that context features such as genotype cluster still contributed meaningfully to the prediction.

**Figure 6:**
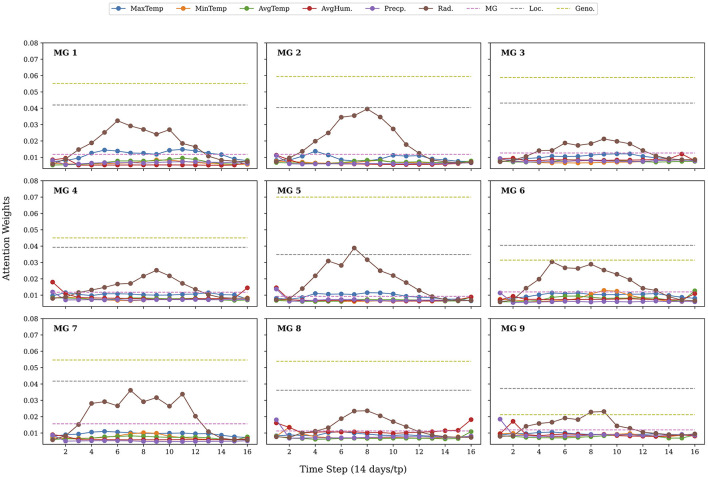
Attention weight across soybean maturity groups (MGs). Radiation related tokens were observed to have the highest values across all MGs, but the next attended tokens differing for the early vs. late MGs, where early MGs emphasized temperature.

To further investigate how the transformer model captured the variable interactions, we analyzed the distribution of attention weights when training and testing across different maturity groups (MGs). Figure 6 illustrates the averaged attention weights for each MG during the growing season. The radiation related tokens were observed to be have the highest attention weights across all MGs. With maximum temperature coming second in earlier-maturing groups (e.g., MG 1–3) assigning greater attention to early-season radiation and temperature, whereas later-maturing groups (MG 7–9) emphasized a mixture of other variables.

## 4 Discussion

This study presents a comprehensive multimodal frameworks for soybean trait prediction that integrates genotypic relatedness, maturity group, and weather features across multiple locations and years in North America. Previous studies have largely relied on narrow environmental ranges with limited years of data, limited number of cultivars, or single modal data. In contrast, our dataset spans diverse maturity zones and climatic conditions encompassing the soybean growing region of North America, and includes thousands of distinct soybean varieties, which is representative of genetic diversity in soybean breeding programs. The transformer-based architecture leveraged the state of the art attention mechanism, capturing temporal dependencies, while simultaneously enabling cross-modal feature learning in this extensive dataset spanning decades, locations, growing conditions and genotypes.

Earlier research in yield prediction primarily focused on meteorological drivers and vegetation indices derived from remote sensing, often using recurrent neural networks or regression-based ensemble models (Xiong et al., 2024; Schwalbert et al., 2020; Shook et al., 2021; Barbosa Dos Santos et al., 2022; Torsoni et al., 2023; Aviles Toledo et al., 2024; Eugenio et al., 2020). These approaches serve a useful purpose; however, they are less generalizable and lack explainability. Studies have worked on predictions for oil and protein in addition to yield, although these have often been in the context for whole field predictions of a single cultivar (Hernandez et al., 2023; Pereyra et al., 2026), and not the performance of different cultivars as in plant breeding. While these previous plant breeding related approaches have achieved moderate success for yield estimation, their extension to seed composition traits such as oil and protein has been more limited due to data scarcity, and imbalance across environments. Herein, the cooperatively run Northern and Southern Uniform Soybean Test are an excellent community resource, and critical for ML models requiring large datasets, such as Transformers.

The transformer framework presented here captures long-range dependencies among environmental, genetic, and management variables, offering a unified architecture that can be adapted to multiple traits. The observed improvements in predictive accuracy show the importance of explicitly modeling spatio-temporal interactions in the data. Crop development and yield formation are inherently time dependent, with physiological processes responding dynamically to environmental and management changes (Hodges and French, 1985; Chen and Wiatrak, 2010). The transformer's temporal attention enables the model to modify weights on critical growth periods, where weather and soil interactions most strongly influence final outcomes. While our models did not utilize soil features as they were unavailable, future studies are needed to incorporate soil variables in yield prediction (Carroll et al., 2024).

Computational approaches, i.e., Transformer-based models, highlighted different environmental variables carrying higher attention weight in predicting yield. For example, both Northern and Southern latitudes had solar radiation and temperature as most predictive. While this is expected and follows physiological reasoning, it is important to note that our models show a high level of accuracy for genotype level yield prediction simply with weather variables and pedigree at individual locations.

Attention-based interpretability revealed that the variables temperature and solar radiation were most attended during the soybean growing season, confirming their central role in regulating photosynthetic activity during seed development (Hamed et al., 2021; Khaki and Wang, 2019). Air temperature has previously reported to affect seed yield, oil, and protein, with higher temperatures often shortening the growing season, thereby reducing yield and oil content (Piper and Boote, 1999; James E. Board and Modali, 2005; Thenveettil et al., 2024), and reducing seed quality (Egli et al., 2005). Solar radiation is also known to affect soybean development, including impacting biomass, seed yield, and seed composition (Shibles and Weber, 1966; Board and Kahlon, 2011; Bianculli et al., 2016). Although we do not interpret the attention weights as absolute measures of variable importance, they reflect the model's focus conditioned on interactions among each variable over each temporal sequence. Our results indicate how the attention weights reflect the importance of environmental data, and that this new method is able to identify and focus on specific variables which have been identified as important in crop physiology. Together, these analyses demonstrate that the model effectively learned physiologically meaningful associations without being implicitly programmed with pre-assigned weights. Additionally, the use of self-attention provides a mechanism to guide future modeling efforts.

The proposed framework advances the state of trait prediction modeling by enabling generalizable, interpretable, and multi-objective inference on genotypes across traits and locations. Nevertheless, challenges remain. Data imbalance across maturity zones and limited representation of extreme weather events may affect performance under uncertain growing weather conditions. Additionally, while attention mechanisms provide interpretability, they remain correlational rather than causal. Thus, there is a need for an integration with domain-based process models for deeper inference. There is a need for incorporating genomic data, such as SNP markers or geno expression profiles, that can enhance genotype-specific predictions and improve the model's ability to capture G × E interactions at a finer resolution. Another promising direction involves integrating other high-throughput phenotyping data such as hyperspectral indices, or canopy temperature to enrich temporal modeling with more physiological context. Both of these approaches would provide greater information on individual genotypes, allowing for greater capture of the variability caused by unique varieties which is presently lacking in this study. However, generating such datasets will take a concerted effort as these types of data are costly and time consuming to generate. Initial advancements are already noted, for example, a recent SNP dataset from the Nothern Uniform Soybean Tests (Wartha et al., 2025).

## 5 Conclusion

In this study, we developed and evaluated a transformer-based deep learning framework for predicting soybean yield across multi-environment trials in the U.S., integrating temporal weather data with genotypic cluster and contextual information. Our approach achieved high predictive performance, with the transformer model outperforming traditional and recurrent baselines in yield prediction. By systematically varying temporal resolutions, we identified that intermediate sampling intervals provided the best balance between temporal fidelity and model efficiency, an insight useful for designing future data embedding strategies in Transformer-based agronomic modeling. We additionally demonstrated that pre-trained temporal representations from yield prediction could be effectively transferred to downstream tasks such as oil and protein prediction, which shows the generalizability of the learned features and highlights a practical strategy for modeling traits with limited labeled data. Leveraging the self-attention mechanism inherent to transformers, our interpretability analysis shows the potential of attention-based models not only as a predictive tool but as a tool to generate actionable insights for breeding and crop management, similar to existing *post-hoc* interpretability methods. This can serve as an important step toward building explicitly interpretable deep learning models, although this should be further tested on the transferability to other crop species.

## Data Availability

The raw data supporting the conclusions of this article will be made available by the authors, without undue reservation.
