# Supplementary material for: Transformer model to determine spatio-temporal relationships of variables, and interpretability for soybean seed yield, oil, and protein prediction
Source: Front Artif Intell. 2026 Feb 26;9:1750108. doi: 10.3389/frai.2026.1750108 (PMC12979532; doi:10.3389/frai.2026.1750108)
Supplement: Supplementary file 1 [file Data_Sheet_1.pdf]

# Supplementary Material

## 1 ELBOW PLOT OF KMEANS ANALYSIS

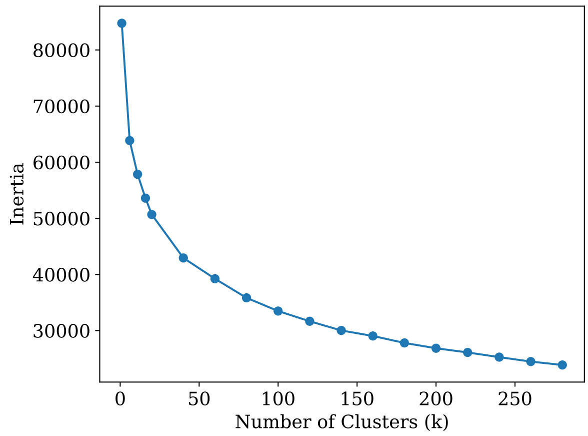


**Figure S1.** KMeans clustering performance across different values of $k$. The curve shows inertia (within-cluster sum of squares), where a sharp decrease followed by a plateau indicates potential ”elbow” points.

## 2 ARCHITECTURE WITH A GLOBAL CONTEXT TOKEN

A variation of the transformer model architecture used for multi-trait prediction in soybean. Input features are segmented and embedded with patch and positional encodings. These are passed through a transformer encoder consisting of stacked layers of multi-head self-attention and feedforward neural networks. The model outputs trait predictions using only the output of the global token, which is passed to a regression head. The model produced a comparable performance to that gotten without the global context token with a $R^2$ Score of 0.769±0.010.

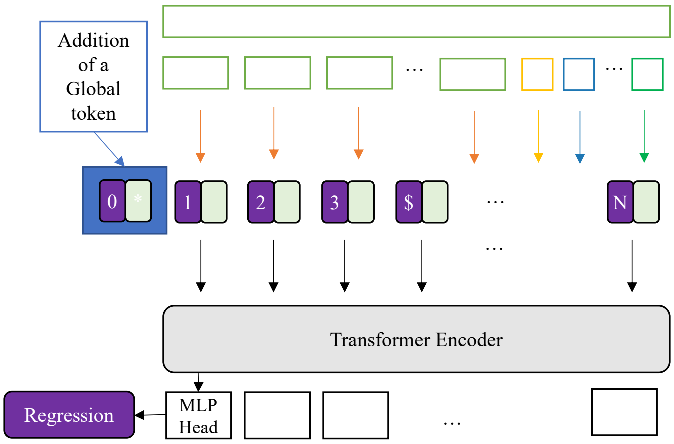


**Figure S2.** A variation of the transformer model architecture that uses a global token for multi-trait prediction in soybean.

# 3 SUPPORT VECTOR REGRESSION HYPERPARAMETER TUNING

**Table S1.** Performance of SVR-RBF with different $\epsilon$ and C values.

| Model | Parameter ($\epsilon$) | C | RMSE | MAE | $R^2$ |
|---|---|---|---|---|---|
| SVR-RBF | 0.005 | 0.1 | 11.469 | 8.934 | 0.403 |
| SVR-RBF | 0.005 | 1 | 8.697 | 6.578 | 0.657 |
| SVR-RBF | 0.005 | 10 | 7.320 | 5.479 | 0.757 |
| SVR-RBF | 0.1 | 0.1 | 11.468 | 8.934 | 0.403 |
| SVR-RBF | 0.1 | 1 | 8.698 | 6.578 | 0.657 |
| SVR-RBF | 0.1 | 10 | 7.319 | 5.478 | 0.757 |

# 4 LASSO HYPERPARAMETER TUNING

**Table S2.** Performance of Lasso regression with varying $\alpha$ values.

| Model | Parameter ($\alpha$) | RMSE | MAE | $R^2$ |
|---|---|---|---|---|
| Lasso | 0.0001 | 7.692 | 5.832 | 0.732 |
| Lasso | 0.001 | 7.809 | 5.940 | 0.723 |
| Lasso | 0.01 | 8.668 | 6.714 | 0.659 |
| Lasso | 0.1 | 10.815 | 8.516 | 0.469 |
| Lasso | 1.0 | 13.576 | 10.798 | 0.164 |

# 5 BIDIRECTIONAL LONG SHORT-TERM MEMORY (BLSTM) HYPERPARAMETER TUNING

**Table S3.** Performance of BLSTM Model with Different Hyperparameters.

| Num Layers | Hidden Size | Learning Rate | RMSE | $R^2$ |
|---|---|---|---|---|
| 2 | 64 | 0.0001 | 7.888 | 0.718 |
| 2 | 512 | 0.0001 | 7.495 | 0.745 |
| 2 | 1024 | 0.0001 | 7.371 | 0.753 |
| 2 | 2048 | 0.0001 | 7.370 | 0.754 |
| 2 | 64 | 0.00001 | 12.073 | 0.339 |
| 2 | 512 | 0.00001 | 7.893 | 0.717 |
| 2 | 1024 | 0.00001 | 7.600 | 0.738 |
| 2 | 2048 | 0.00001 | 7.597 | 0.738 |
| 4 | 64 | 0.0001 | 7.845 | 0.721 |
| 4 | 512 | 0.0001 | 7.430 | 0.750 |
| 4 | 1024 | 0.0001 | 7.426 | 0.750 |
| 4 | 2048 | 0.0001 | 7.417 | 0.750 |
| 4 | 64 | 0.00001 | 11.387 | 0.412 |
| 4 | 512 | 0.00001 | 7.601 | 0.738 |
| 4 | 1024 | 0.00001 | 7.481 | 0.746 |
| 4 | 2048 | 0.00001 | 7.419 | 0.750 |
| 8 | 64 | 0.0001 | 7.981 | 0.711 |
| 8 | 512 | 0.0001 | 13.948 | 0.117 |
| 8 | 1024 | 0.0001 | 13.984 | 0.113 |
| 8 | 2048 | 0.0001 | 13.993 | 0.112 |
| 8 | 64 | 0.00001 | 10.637 | 0.487 |
| 8 | 512 | 0.00001 | 7.600 | 0.738 |
| 8 | 1024 | 0.00001 | 7.490 | 0.745 |
| 8 | 2048 | 0.00001 | 7.470 | 0.747 |

# 6 BIDIRECTIONAL RECURRENT NEURAL NETWORKS (BRNN) HYPERPARAMETER TUNING

**Table S4.** BRNN performance across various configurations for Yield.

| Hidden Size | Num Layers | Learning Rate | RMSE | $R^2$ |
|---|---|---|---|---|
| 2 | 64 | 0.0001 | 7.816 | 0.723 |
| 2 | 512 | 0.0001 | 7.393 | 0.752 |
| 2 | 1024 | 0.0001 | 7.433 | 0.749 |
| 2 | 2048 | 0.0001 | 7.742 | 0.728 |
| 2 | 64 | 0.00001 | 13.448 | 0.179 |
| 2 | 512 | 0.00001 | 7.699 | 0.731 |
| 2 | 1024 | 0.00001 | 7.541 | 0.742 |
| 2 | 2048 | 0.00001 | 7.429 | 0.750 |
| 4 | 64 | 0.0001 | 7.462 | 0.747 |
| 4 | 512 | 0.0001 | 7.407 | 0.751 |
| 4 | 1024 | 0.0001 | 8.317 | 0.686 |
| 4 | 2048 | 0.0001 | 13.910 | 0.122 |
| 4 | 64 | 0.00001 | 11.634 | 0.386 |
| 4 | 512 | 0.00001 | 7.500 | 0.745 |
| 4 | 1024 | 0.00001 | 7.393 | 0.752 |
| 4 | 2048 | 0.00001 | 7.432 | 0.749 |
| 8 | 64 | 0.0001 | 7.452 | 0.748 |
| 8 | 512 | 0.0001 | 13.989 | 0.112 |
| 8 | 1024 | 0.0001 | 13.982 | 0.113 |
| 8 | 2048 | 0.0001 | 13.993 | 0.112 |
| 8 | 64 | 0.00001 | 9.635 | 0.579 |
| 8 | 512 | 0.00001 | 7.506 | 0.744 |
| 8 | 1024 | 0.00001 | 7.459 | 0.748 |
| 8 | 2048 | 0.00001 | 7.421 | 0.750 |

# 7 PREDICTION RMSE AND MAE TRENDS ACROSS TEMPORAL RESOLUTIONS FOR YIELD, OIL, AND PROTEIN

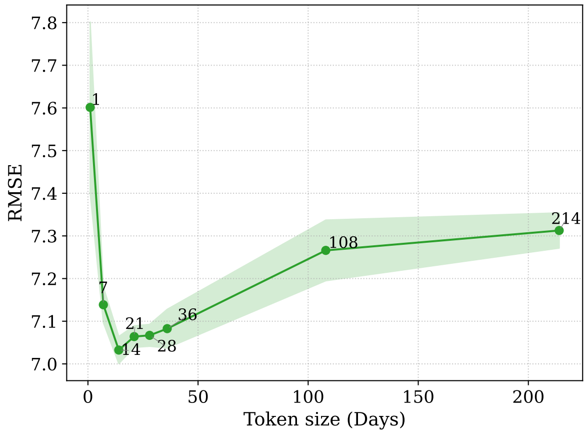


(a) Yield–RMSE

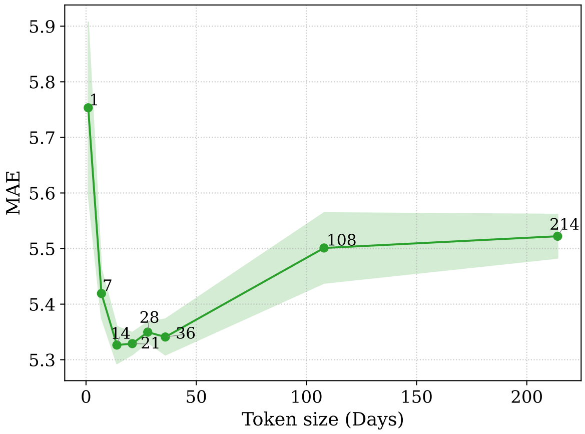


(b) Yield–MAE

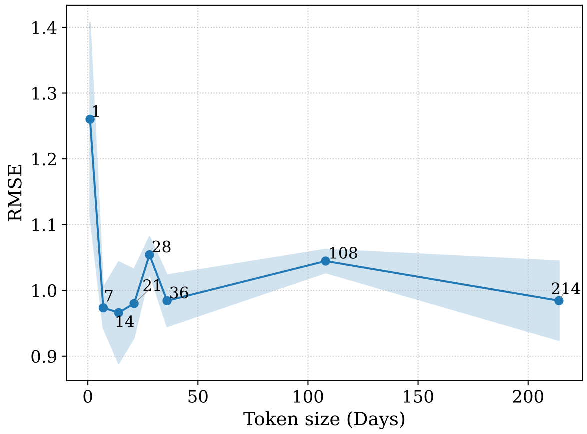


(c) Oil–RMSE

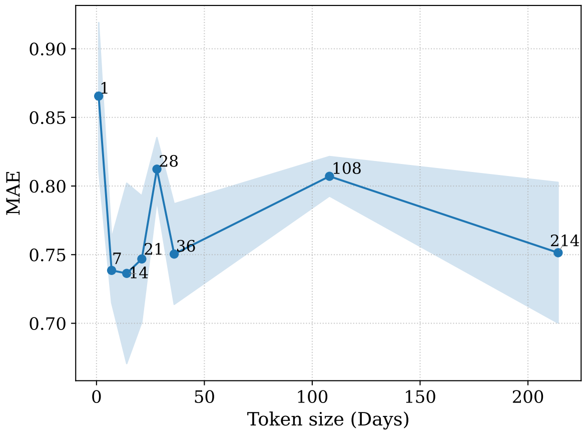


(d) Oil–MAE

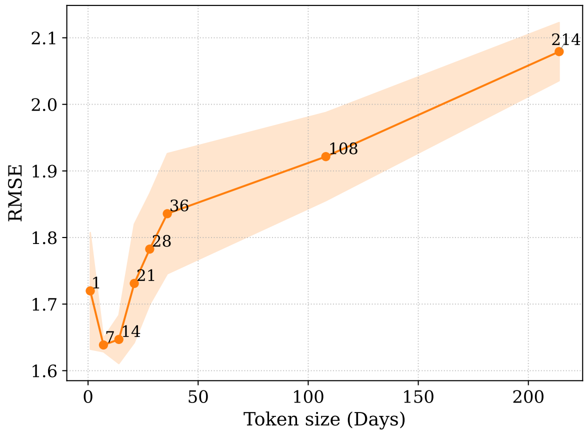


(e) Protein–RMSE

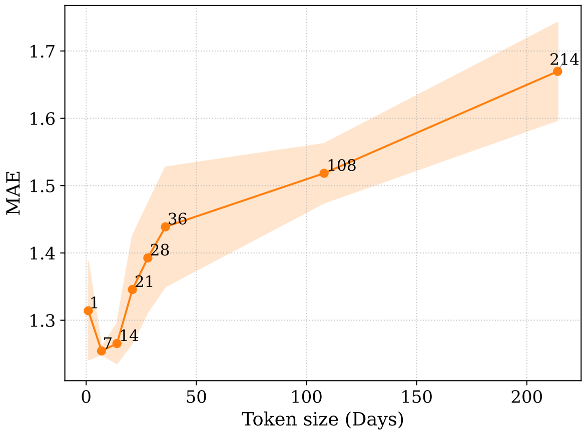


(f) Protein–MAE

**Figure S3.** RMSE and MAE prediction performance across temporal resolutions for yield, oil, and protein. Panels (a)–(f) correspond to RMSE and MAE for each trait.

# 8 PER-SAMPLE AOPCR-BASED FAITHFULNESS EVALUATION USING RANDOMLY PICKED SAMPLES.

**Table S5.** Per-sample AOPCR-based faithfulness evaluation. Bold indicates best (ties allowed); underline indicates second-best. Values shown to 3 decimal places.

| Sample | Method | Comprehensiveness (↑) | | Sufficiency (↓) | |
|---|---|---|---|---|---|
| | | MAE | MSE | MAE | MSE |
| 409 | Attention | **0.343** | **4.653** | **0.291** | <u>3.548</u> |
| | DeepLIFT | 0.282 | 3.234 | 0.325 | 4.410 |
| | Gradient SHAP | 0.312 | 3.976 | 0.318 | 4.114 |
| | Integrated Gradients | <u>0.340</u> | <u>4.648</u> | <u>0.292</u> | **3.536** |
| | LIME | 0.062 | 0.163 | 0.425 | 7.103 |
| 1424 | Attention | <u>0.308</u> | <u>3.843</u> | 0.338 | 4.583 |
| | DeepLIFT | 0.285 | 3.335 | 0.365 | 5.231 |
| | Gradient SHAP | **0.315** | **4.165** | **0.282** | **3.364** |
| | Integrated Gradients | 0.288 | 3.506 | <u>0.335</u> | <u>4.448</u> |
| | LIME | 0.069 | 0.222 | 0.421 | 6.928 |
| 1824 | Attention | **0.340** | <u>4.567</u> | **0.289** | **3.489** |
| | DeepLIFT | 0.299 | 3.539 | 0.331 | 4.413 |
| | Gradient SHAP | <u>0.335</u> | **4.575** | 0.296 | 3.664 |
| | Integrated Gradients | <u>0.335</u> | 4.455 | <u>0.292</u> | <u>3.561</u> |
| | LIME | 0.068 | 0.209 | 0.420 | 6.879 |
| 2286 | Attention | <u>0.295</u> | <u>3.672</u> | **0.310** | **3.988** |
| | DeepLIFT | 0.209 | 2.018 | 0.366 | 5.292 |
| | Gradient SHAP | 0.197 | 1.835 | 0.376 | 5.540 |
| | Integrated Gradients | **0.314** | **4.048** | <u>0.337</u> | <u>4.596</u> |
| | LIME | 0.061 | 0.166 | 0.426 | 7.137 |
| 8935 | Attention | **0.316** | <u>3.987</u> | **0.305** | **3.855** |
| | DeepLIFT | 0.164 | 1.401 | 0.384 | 5.809 |
| | Gradient SHAP | 0.245 | 2.823 | 0.359 | 5.116 |
| | Integrated Gradients | **0.316** | **4.074** | <u>0.328</u> | <u>4.437</u> |
| | LIME | 0.064 | 0.171 | 0.423 | 6.990 |
| 9674 | Attention | <u>0.298</u> | <u>3.897</u> | **0.321** | **4.275** |
| | DeepLIFT | 0.284 | 3.380 | 0.357 | 5.078 |
| | Gradient SHAP | 0.290 | 3.611 | <u>0.322</u> | <u>4.286</u> |
| | Integrated Gradients | **0.310** | **3.928** | 0.344 | 4.788 |
| | LIME | 0.063 | 0.168 | 0.425 | 7.090 |

# 9 AVERAGE ATTENTION WEIGHTS OF YIELD PREDICTIVE VARIABLES ACROSS TEST SET RUNS FOR THE VARIOUS SAMPLING INTERVALS

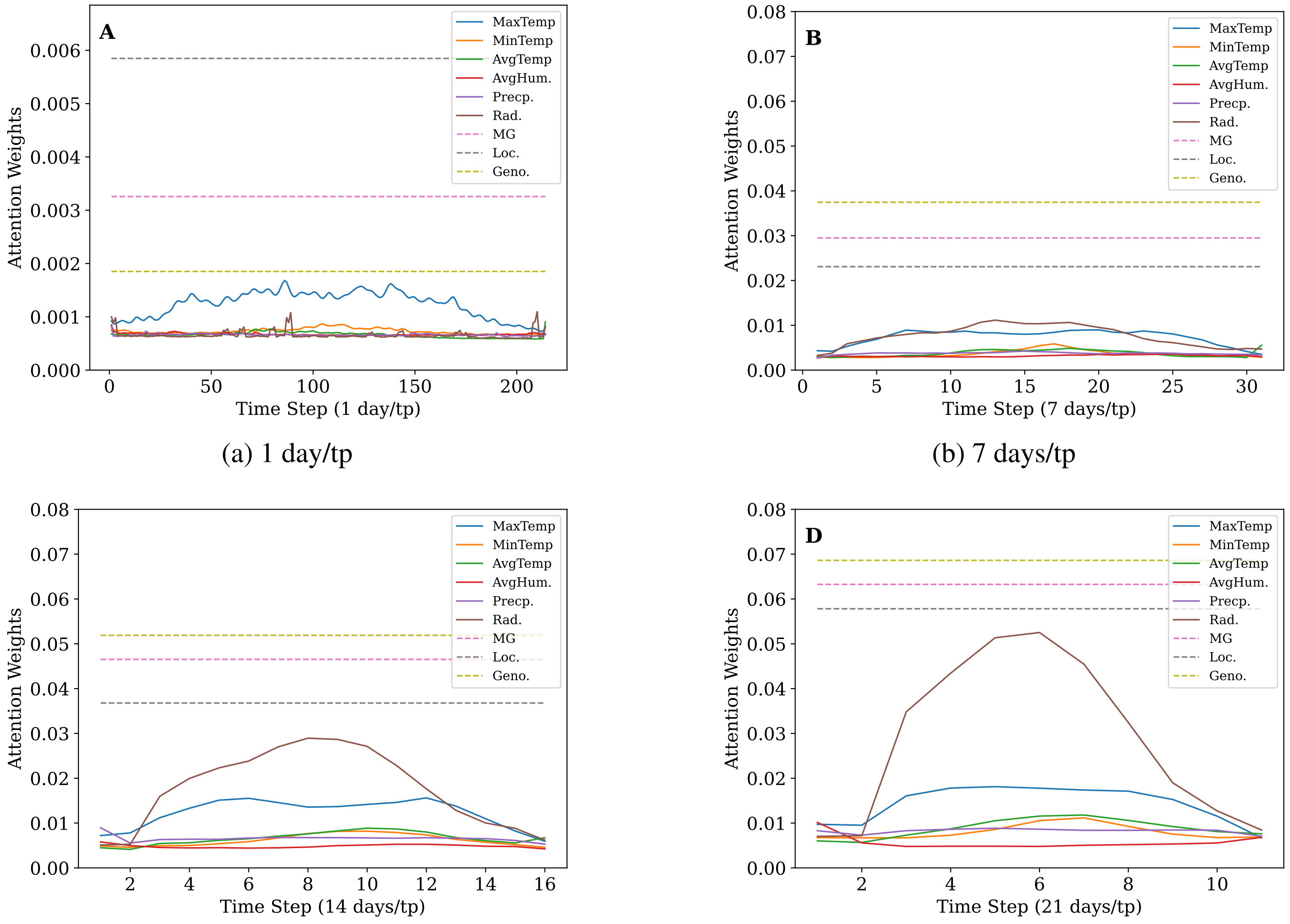


(a) 1 day/tp

(b) 7 days/tp

(c) 14 days/tp

(d) 21 days/tp

**Figure S4.** Attention weights of yield-predictive variables for sampling frequencies of (a) 1, (b) 7, (c) 14, and (d) 21 days per timestep.

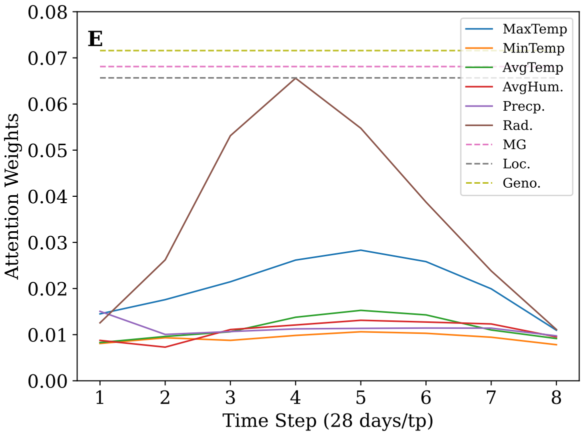


(e) 28 days/tp

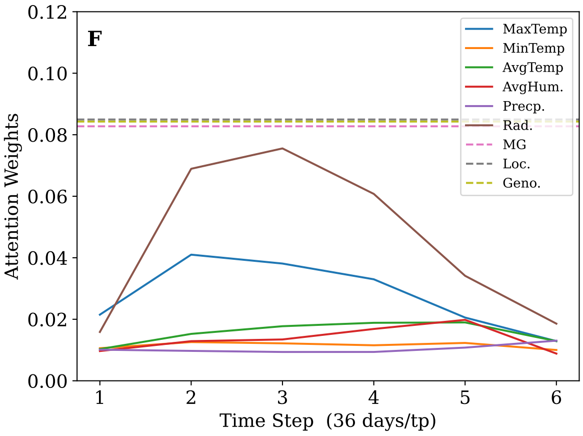


(f) 36 days/tp

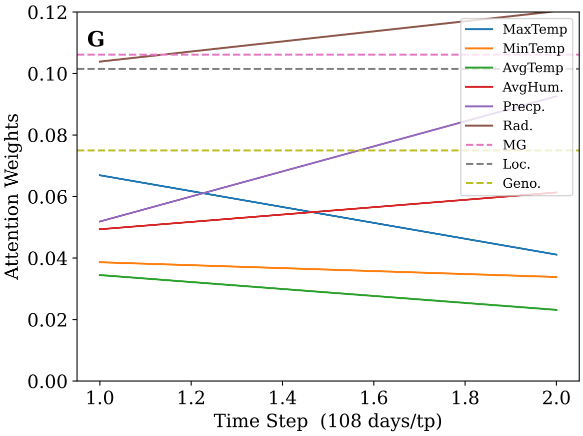


(g) 108 days/tp

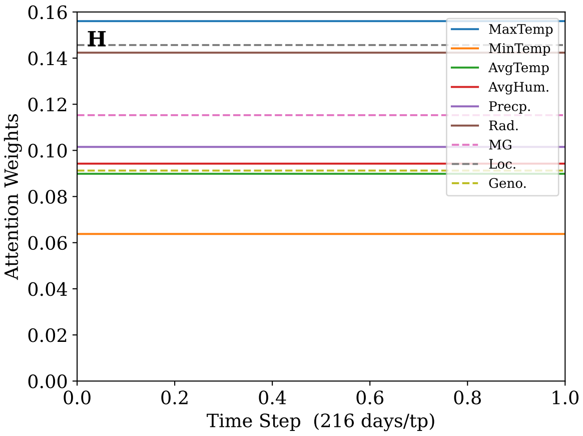


(h) 216 days/tp

**Figure S5.** Attention weights of yield-predictive variables for sampling frequencies of (e) 28, (f) 36, (g) 108, and (h) 216 days per timestep.

# 10 AVERAGE ATTENTION WEIGHTS OF YIELD PREDICTIVE VARIABLES FOR TWO RANDOM TIMESERIES IN THE TEST DATASET FOR 7 AND 14 DAYS/TP SAMPLING INTERVAL

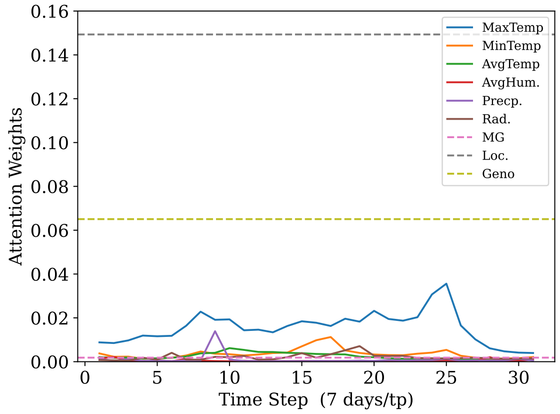


(a) 7-day sampling

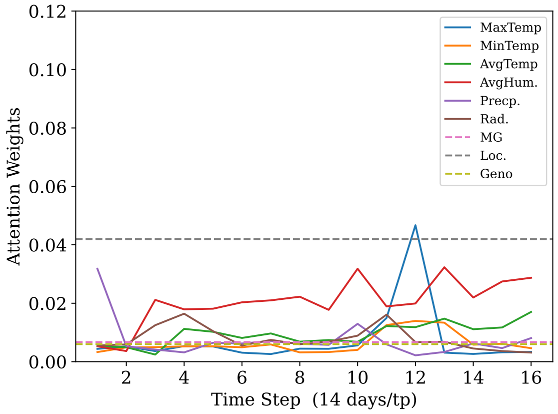


(b) 14-day sampling

**Figure S6.** Attention weights of yield-predictive variables for two random test-set time series at (a) 7-day and (b) 14-day token sampling intervals.

# 11 RMSE AND MAE OF OIL AND PROTEIN PREDICTION FOR FULLY FINE-TUNED ENCODER VS FROZEN ENCODER

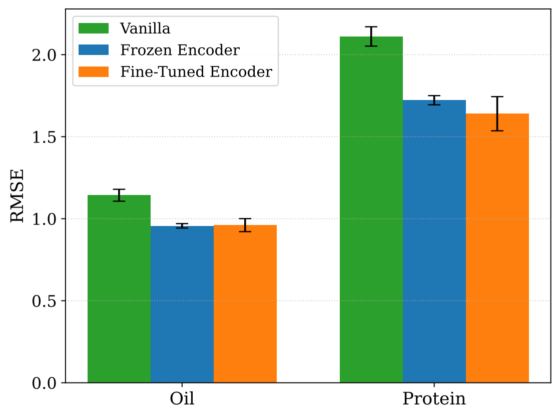


(a) RMSE

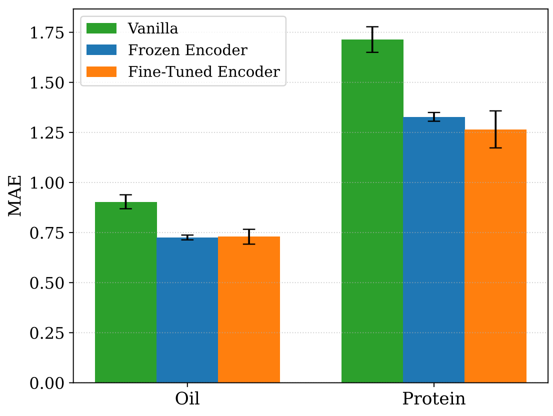


(b) MAE

**Figure S7.** Oil and protein prediction performance measured using (a) RMSE and (b) MAE.

# 12 EFFECT OF GENOTYPE, WEATHER, AND MANAGEMENT ON YIELD PREDICTION PERFORMANCE.

**Table S6.** Effect of genotype, weather, and management on yield prediction performance. Weather modality tokenized at 14 days/tp interval

| Input Configuration | $R^2$ Score | RMSE | MAE |
|---|---|---|---|
| Geno | 0.043±0.002 | 14.520±0.019 | 11.534±0.019 |
| Geno × Weather | 0.724±0.006 | 7.795±0.082 | 5.858±0.083 |
| Geno × Weather × MG | 0.771±0.003 | 7.098±0.049 | 5.369±0.048 |
